# Pesticide Research on Environmental and Human Exposure and Risks in Sub-Saharan Africa: A Systematic Literature Review

**DOI:** 10.3390/ijerph19010259

**Published:** 2021-12-27

**Authors:** Samuel Fuhrimann, Chenjie Wan, Elodie Blouzard, Adriana Veludo, Zelda Holtman, Shala Chetty-Mhlanga, Mohamed Aqiel Dalvie, Aggrey Atuhaire, Hans Kromhout, Martin Röösli, Hanna-Andrea Rother

**Affiliations:** 1 Swiss Tropical and Public Health Institute (Swiss TPH), 4002 Basel, Switzerland; adrianaveludo@gmail.com (A.V.); shalachetty7@gmail.com (S.C.-M.); martin.roosli@swisstph.ch (M.R.); 2 Faculty of Science, University of Basel, 4002 Basel, Switzerland; 3 Institute for Risk Assessment Sciences (IRAS), Utrecht University, 3584 CM Utrecht, The Netherlands; elodie.blouzard96@gmail.com (E.B.); h.kromhout@uu.nl (H.K.); 4 Department of Environmental Systems Science, ETH Zurich, 8092 Zurich, Switzerland; chenjie_wan@outlook.com; 5 Centre for Environmental and Occupational Health Research, School of Public Health and Family Medicine, University of Cape Town, Cape Town 7729, South Africa; zeldaholtman57@gmail.com (Z.H.); aqiel.dalvie@uct.ac.za (M.A.D.); andrea.rother@uct.ac.za (H.-A.R.); 6 Division of Environmental Health, School of Public Health and Family Medicine, University of Cape Town, Cape Town 7729, South Africa; 7 Uganda National Association of Community and Occupational Health (UNACOH), Kampala 12590, Uganda; atuagrey3@gmail.com

**Keywords:** agriculture, environmental risks, exposure risks, food production, organochlorine, organophosphate, pesticides, plant protection products, public health, SDG 2, SDG 12, SSA

## Abstract

On the African continent, ongoing agriculture intensification is accompanied by the increasing use of pesticides, associated with environmental and public health concerns. Using a systematic literature review, we aimed to map current geographical research hotspots and gaps around environmental and public health risks research of agriculture pesticides in Sub-Saharan Africa (SSA). Studies were included that collected primary data on past and current-used agricultural pesticides and assessed their environmental occurrence, related knowledge, attitude and practice, human exposure, and environmental or public health risks between 2006 and 2021. We identified 391 articles covering 469 study sites in 37 countries in SSA. Five geographical research hotspots were identified: two in South Africa, two in East Africa, and one in West Africa. Despite its ban for agricultural use, organochlorine was the most studied pesticide group (60%; 86% of studies included DDT). Current-used pesticides in agriculture were studied in 54% of the study sites (including insecticides (92%), herbicides (44%), and fungicides (35%)). Environmental samples were collected in 67% of the studies (e.g., water, aquatic species, sediment, agricultural produce, and air). In 38% of the studies, human subjects were investigated. Only few studies had a longitudinal design or assessed pesticide’s environmental risks; human biomarkers; dose-response in human subjects, including children and women; and interventions to reduce pesticide exposure. We established a research database that can help stakeholders to address research gaps, foster research collaboration between environmental and health dimensions, and work towards sustainable and safe agriculture systems in SSA.

## 1. Introduction

In Sub-Saharan Africa (SSA), agriculture represents an economically important sector, contributing to the livelihoods of rural families and employing the biggest and still growing workforce [1]. Agriculture production systems are at the same time under pressure due to climate change [2], the need to produce about double the amount of food by 2050 [3], and to achieve some of the sustainable development goals (SDGs) [4]. Therefore, a “new green revolution” has been propagated, which is resulting in substantial land-use change and intensification [5,6]. The transition of these mostly smallholder farms to larger conventional agricultural entities requires additional agrochemical inputs [7]. Across all 46 countries in SSA, the Food and Agriculture Organization of the United Nations (FAO) estimated that pesticide use increased by 1.5 times between 2006 and 2019, reaching more than 100 kg tons per year [8]. Despite being banned in Western countries, several persistent and highly hazardous insecticides (such as organochlorines (OCPs), organophosphates and carbamates) are still frequently used and exported to SSA [9,10]. Overall, this leads to situations where there are hundreds of different current-used pesticide (CUP) formulations in use, consisting of multiple active ingredients [11] with established environmental and public health effects [12,13]. This results in exposure to pesticide mixtures that can act cumulatively or synergistically on non-target organisms, including humans [14]. At the same time, there is an ongoing shift towards agriculture that uses more herbicides and fungicides (e.g., due to no-tillage farming [15] or greenhouse-based horticulture systems, such as flower farming [16]). There are gaps in pesticide use data (e.g., no current data exists for Nigeria [8]) or assumptions are based on generic sales data (e.g., in South Africa [11,17]). Outdated information on pesticide use creates misinformation on current agriculture practices and related risks.

There are only a few research studies on environmental occurrence [18,19] or human exposure and health outcomes of pesticides [20] in low- and middle-income countries (LIMCs), with particular research gaps in SSA. Additionally, research studies in LMICs often have a disciplinary focus on a specific environmental matrix (e.g., water [21], soil [22], or dust [23]); knowledge, attitude, and practice (KAP) of users [16,24,25]; human exposure; and health risks for farmers and farmworkers and vulnerable groups, such as women and children [24,25]. A context-specific overview or an integration of findings across research studies from different matrices and population groups is therefore often missing. However, such an overview would be valuable to inform and guide possible interventions to reduce environmental and public health risks. In light of the expanding agriculture sector and the growing and aging population in SSA, an integrated understanding and monitoring of pesticide pollution combining information from environmental, food, and health systems are therefore needed to achieve several of the SDGs [26]. Hence, efforts should be undertaken to build interdisciplinary research networks, which can exchange information and build cross-discipline interventions capacity towards promoting health and environmental risk reduction during pesticide use.

This paper aims to provide an overview of recent research on legacy and current-used agriculture pesticides and their environmental and human health issues while highlighting research advances and gaps in SSA. We therefore conducted a systematic review to identify studies that collected primary data on pesticide’s environmental occurrence (e.g., in water, air, or soil), KAP of applicators, and human environmental and occupational exposure and health outcomes in SSA. The following four research questions were asked: (i) where are geographical pesticide research hotspots in SSA? (ii) Which are the priority pesticides investigated? (iii) What are the general characteristics of the human subjects and environmental samples studied? And (iv) who are the authors and institutions driving pesticide research in SSA?

This paper is part of the “Africa Pesticide Intervention Studies” (APSENT) project. APSENT aims to build and sustain a network around researchers focusing on pesticides and subsequently interventions to reduce risks for the environment and human health across Africa.

## 2. Materials and Methods

### 2.1. Search Strategy

We conducted a systematic literature review to generate an overview of research studies reporting on past and current-used agriculture pesticides involving human subjects or environmental samples in any of the 46 countries in SSA over the past 16 years (between 1 January 2006 till 12 October 2021). The review was done following the PRISMA guidelines [27]. Peer-reviewed original research papers were searched in English, French, Spanish, and Portuguese in the PubMed and the Web of Science databases. The search included different search terms for pesticides (e.g., plant protection product*, herbicide*; total 11 search terms) and all 46 countries in SSA, including different spellings in their national language (e.g., Africa*, Angola*; total 66 terms). Matching terms for human subjects were used as follows (objective exposure markers (e.g., urine, blood; total 21 terms); qualitative exposure (e.g., questionnaire*, KAP; total five terms); and exposure groups (farm*, children*; total nine terms)). In addition, terms for environmental samples were included (e.g., residual*, food; total seven terms). Finally, studies were excluded if they included the term malaria or helminth* to exclude studies that were exclusively on human disease vector control. This reduced the number of titles from 14,341 to 10,470. The full search syntax can be found in the Supplementary Materials Table S1.

### 2.2. Article Selection

Title and abstract screening of retrieved articles were performed by four research assistants (W.C. and E.B. literature 2006–2016; Z.H. and A.V. literature 2016–2021). The selected articles for full-text review were also reviewed by the leading author of this manuscript (S.F.). The inclusion criteria were peer-reviewed original studies describing primary data on pesticides in the environment or from human subjects. Studies were excluded if they were from outside of SSA, reviews, described a sampling or laboratory method, or discussed only human disease vector control (e.g., malaria or helminths). Included articles were screened for eligibility a second time by the research assistants and S.F. All articles were stored in Mendeley (version 2.59.0, Elsevier, London, UK).

### 2.3. Data Extraction

For each article that reported on more than one study site (i.e., in a different country or sites that are more than 1000 km apart), a separate entry/extraction was made. The following data were extracted from all identified studies sites:

*General study characteristics:*

- Year of publication, year of data collection, and over how many years the data were collected
- Country where the study was conducted, the region where the study was conducted (when more than one region only one was reported (i.e., the first one which was mentioned)) and GPS coordinates of the study site (if the specific site was not mentioned, a random GPS point in the region was taken; in case no region was stated, the GPS of the capital city of the country was taken).

*Pesticide investigated:*

- Any organochlorine pesticides (OCPs), dichlorodiphenyltrichloroethane (DDT),
- Any current-used pesticides (CUP; all pesticides other than OCPs), type of CUP (herbicides, fungicides, insecticides), chemical group of CUP insecticides (organophosphates, pyrethroids or others), and if no pesticides were mentioned by type or chemical group.

*Environmental samples:*

- Information on the matrices collected (e.g., water, soil, or air)
- Health risks assessment conducted for consumption, ingestion, inhalation, or dermal exposure.
- Environmental risk assessment conducted.

*Human subjects:*

- Study design (i.e., intervention study, cross-sectional, longitudinal, retrospective, and case report)
- Study population (i.e., general population or occupational population)
- Sex of participants
- Number of individuals in the study population
- Human health outcomes: human health outcome group (e.g., signs and symptoms of acute poisoning, respiratory health) and outcome diagnosis method (i.e., objective measures, self-reported, or doctor-diagnosed)
- Human pesticide exposure: self-reported exposure, exposure algorithm, objective exposure marker (e.g., biomarkers in urine or blood and active ingredients in wristband)
- KAP and training of pesticide user

### 2.4. Analysis

All extracted data from the 391 articles and 469 study sites are available in the Supplementary Materials Table S2 as an excel database. To indicate the geographical hotspots of the identified study sites, GPS locations were mapped, and a density heat map was produced using QGSI (version 3.4.4-Madeira, QGIS Development Team, available online: www.QGIS.org (accessed on 28 October 2018)). To identify the most researched pesticides, a frequency table for OCPs, CUPs, most studied types, and chemical groups was created. To indicate integrated and individual assessments between environmental and human studies, a Venn diagram was produced showing proportional overlaps between studies reporting on human subjects, environmental samples, human outcome and risk assessment, and environmental risk assessment. To generate a global overview of the extracted data, frequency tables are reported for all above-described parameters from studies collecting environmental samples and assessing human subjects across all study entries.

To observe differences between countries, a stratified frequency analysis of the most relevant parameters is also provided for each identified country (i.e., for human exposure, human health, KAP, intervention studies, OCP, and CUP). To indicate if the reported data reflect the current situation at the date of publication, the time between data collection and publication was assessed. In case the date of collection was not provided, the article was excluded from this analysis.

Finally, to identify important researchers in the field, a summary table of the authors’ contributions as first, second, last, and as the corresponding author is provided. All statistical analyses were conducted in R (version 3.6.3, RStudio version 1.2, Foundation for Statistical Computing, Boston, MA, USA).

## 3. Results

### 3.1. Identified Research Articles

**Figure 1:**
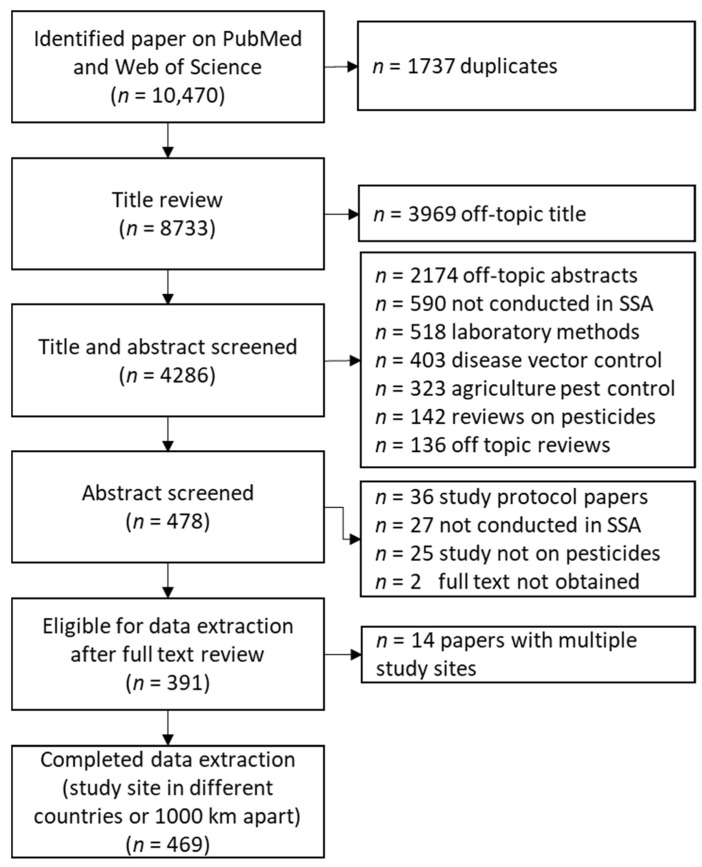
Flow chart indicating the number of articles hits during the literature review process, paper identification, and data extraction.

The review in Pubmed and Web of Science resulted in 10,470 articles, out of which 1737 were duplicates, leaving 8733 for the title and abstract review (Figure 1). The title and abstract review resulted in 4286 articles for detailed abstract review, out of which 478 articles were identified for full-text review after meeting the inclusion criteria. Papers not included in the review were clustered into the following categories: study not conducted in SSA (590), laboratory method development or validation of pesticides (518), disease vector control (403), agriculture pest management (323), reviews on pesticides (142), and reviews off topic, such as agriculture practices (136). Out of the full-text reviews, 391 fulfilled the eligibility criteria. Articles were excluded for the following reasons: only describing a study design protocol (36), study not conducted in SSA (27), study on other chemicals than pesticides (25), and full text not obtained (2). Thirteen studies reported on more than one country, resulting in 469 individual study sites for which we extracted data separately; all extracted data are reported in Supplementary Materials Table S2.

### 3.2. Spatial Distribution of the Research Studies across Sub-Saharan Africa

**Figure 2:**
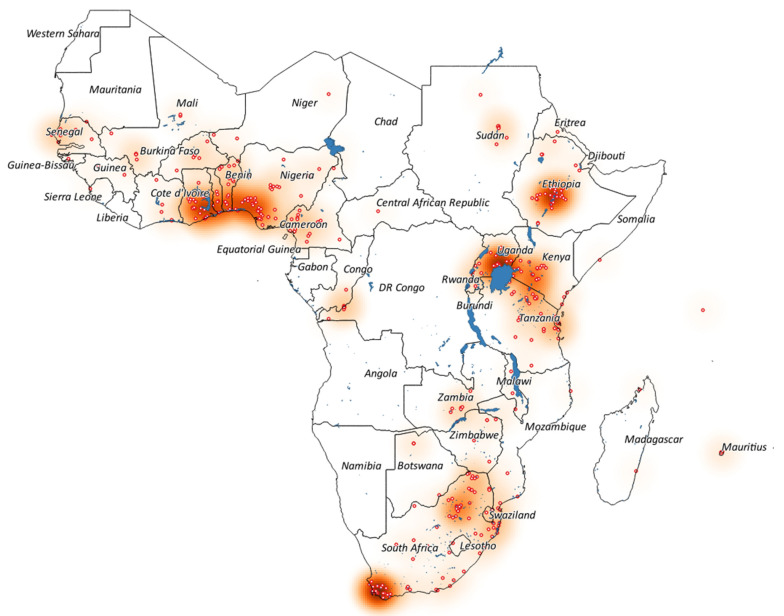
Map of Sub-Saharan Africa, indicating all 469 study sites (red circles) within 37 countries out of 46, which were included in the 391 identified papers between 2006 and 2021. Intense red on the heat map indicates an increased density of study sites; blue areas indicate water bodies.

The 469 study sites were located in 37 countries, while for 12 countries, no study could be found (Figure 2, Supplementary Materials Table S3). Most studies were conducted in South Africa (102 (22%)), followed by Nigeria (45 (10%)), Ethiopia (42 (9%)), and Tanzania and Uganda (both 37 (8%)). The four intervention studies were conducted in South Africa (two studies), Uganda, and Mali.

Based on the extracted GPS coordinates of the study sites, five distinct regional hotspots could be identified where most studies were conducted: (i) in South Africa around the Western Cape, which is dominated by research on export-oriented fruit and wheat farming systems; (ii) in the northeast of South Africa, which focused mostly on smallholder farming systems combined with pesticide use for vector control; (iii) the region around Lake Victoria in Uganda and Tanzania and Lake Naivasha in Kenya, dominated by smallholder and flower farming systems; (iv) the Ethiopian Rift Valley lakes, where studies were mostly conducted on flower, vegetable, and smallholder farming systems; and (v) in West Africa between Ghana, Togo, Benin, and Nigeria, with a wide focus on smallholder and larger-scale farming systems.

### 3.3. Pesticides Investigated

Most of the 469 study sites included an assessment of OCPs (281 (60%)), while the majority focused on DDT (241 (86%)) (Table 1). CUPs were studied in half of the studies (253 (54%)), and 101 (22%) investigated OCPs and CUPs together, and 8% of the studies did not specify the assessed pesticides. Overall, the CUP studies, 68 (27%) investigated mixtures of insecticides, fungicides, and herbicides. Most CUPs were insecticides (233 (92%)), followed by herbicides (110 (44%)) and fungicides (89 (35%)). Among the CUP insecticides, organophosphates (180 (77%)) and pyrethroids (106 (46%)) were the most studied chemical groups.

### 3.4. Extracted Human Subject and Environmental Sample Information

**Figure 3:**
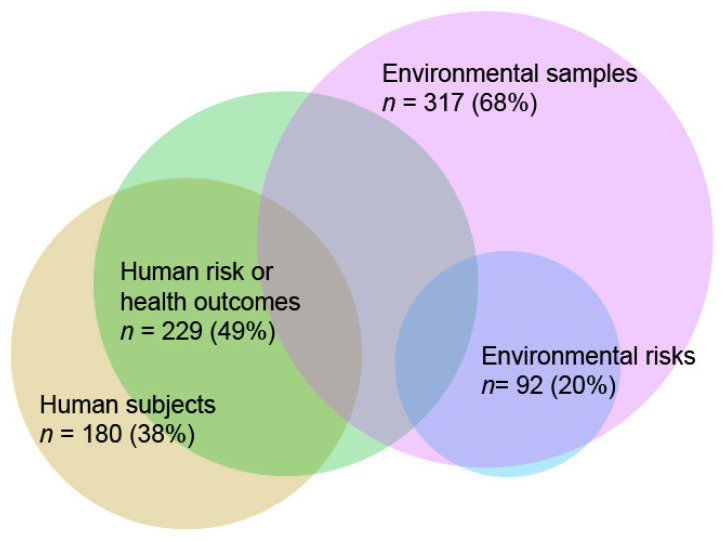
Venn diagram (number of study sites (%)) showing the proportional overlaps between environmental samples, human subjects (i.e., involving any human participants), health outcomes and risks and environmental risk assessments (*n* = 469).

Of the 469 entries, 6% of the studies included both environmental samples and human subjects, 61% included only environmental samples, and 32% studied only human subjects (Figure 3 Fewer than than half of the study sites, human health outcomes or risks were assessed (49%), while environmental risks were assessed only in 20% of the sites.

### 3.5. Human Subjects

A total of 180 (38%) study sites investigated human subjects (Table 2), out of which four (2%) conducted an intervention on how to reduce pesticide use or exposure, 123 (68%) assessed health outcomes, 49 (27%) did only an exposure assessment, and four (2%) just included a KAP assessment. Occupational populations were assessed the most (108 (60%)), followed by general populations (e.g., resident populations; (40 (22%)), both occupational and environmental populations (29 (16%)), and self-inflicted poisoning (3 (2%)). The survey design was predominately cross-sectional (151 (84%)). Only a few prospective longitudinal studies (18 (10%)), retrospective studies (6 (3%)), and case reports (5 (3%)) were conducted. Data were collected from adult populations (164 (91%)), while studies including children were only a few (16 (9%)). In most studies, both sexes were participating (111 (62%)). However, in most of these studies, women were represented only in a marginal proportion of the total study population (data not extracted). Sex-specific studies were done twice as often with men (39 (22%)) than with women (20 (11%)). In ten studies, the sex of the participant was not reported. The number of participants was highest in retrospective studies (median (range)) (797 (96-7427)), followed by prospective longitudinal studies (310 (15-1461)) and cross-sectional studies (183 (7-1496)).

#### 3.5.1. Human Health Outcomes

At 127 study sites, health outcomes were assessed, dominated by descriptive reporting or model-based risk assessments (91 (72%)), while only 36 (28%) assessed exposure-outcome relationships with regression models (Table 2). Interestingly, 28 (78%) these regression models indicated a significant associations towards a worse health outcome. The health outcomes in the 127 study sites could be stratified into ten broader categories out of which “signs and symptoms of acute poisoning” (38 (30%)) were most assessed, followed by “doctor-diagnosed pesticide poisoning” (24 (19%)) and neurological (17 (11%)), reproductive (14 (9%)), and respiratory (12 (9%)) health problems. The majority of the health assessments were self-reported (58 (46%)) (i.e., signs and symptoms of acute poisoning in questionnaire interviews), followed by objective measured exposure markers (41 (32%)) (e.g., acetylcholinesterase (AChE) measurement in the blood), doctor-diagnosed (16 (13%)) (e.g., pesticide poisoning), and model-based (12 (9%)) (e.g., calculating health burden based on urine biomarkers).

#### 3.5.2. Human Exposure and KAP of Pesticide Use

Most of the 127 study sites including human subjects assessed pesticide exposure (171 (95%)). The majority assessed only self-reported exposure (i.e., via questionnaire interviews (107 (63%))). More specific exposure assessment was done in a third of the studies, i.e., 60 studies (35%) collected objective exposure data, while only 2% (4 studies) estimated cumulative exposure via a context-specific exposure algorithm. Objective exposure markers were assessed in blood (36 (60%)), urine (14 (23%)), wristbands (5 (8%)), human breast milk (4 (7%)), and other matrices (5 (8%)) (e.g., hair, body patches).

KAP was assessed in several studies alongside health or exposure assessments (149 (83%)). Among the KAP studies, 40 studies (27%) also assessed KAP if the farmers had attended training on pesticide use.

### 3.6. Environmental Samples

Among 317 (68%) studies reporting on environmental samples (Table 3), most sampled one specific matrix (e.g., water or soil) (216 (68%)). Fifty-five studies reported on two matrices (17%) and 45 on three or more (14%). Water samples were collected the most (93 (30%)), followed by aquatic species (79 (25%)), sediment (73 (23%)), agricultural produce (72 (23%)), air (57 (18%)), soil (39 (12%)), dust (2 (1%)), and other matrices (34 (10%), e.g., livestock, plants).

Based on the measured pesticides in the environment, 89 (28%) undertook an environmental risk assessment; in most of these risk assessments, at least one detected pesticide level was above a risk threshold (76 (85%)). Most environmental risk assessment were done based on pesticide levels measured in water (41 (44%)), followed by sediment (33 (45%)), aquatic species (28 (34%)), and agriculture produce (24 (33%)).

Human health risk assessment based on 112 (35%) studies sampled an environmental matrix, indicating half of the studies’ levels were above human health risk thresholds (53 (47%)). The measured pesticide levels were mostly assessed for unspecific human health risks (78 (70%)), followed by cancer (14 (13%)), pesticide poisoning (13 (12%)), and reproductive health issues (5 (5%)). Human consumption of the environmental matrix was the most assessed exposure pathway (107 (96%)).

### 3.7. Duration and Temporal Distribution of Data Collection

Studies were published on average 3.8 years after the data were collected (range 0 to 18 years) based on 307 studies. Eighty-one studies did not report the year of data collection. Almost a third of these articles (117 (30%)) reported on data collected over more than one year; on average, data were collected over 3.7 years (range 2 to 20 years).

### 3.8. Authors and Research Institutions

Authors in a leading position (first, second, or last) authoring most frequently were: MA. Dalvie and L. London (South Africa, both *n* = 14), H. Bouwman (South Africa; *n* = 10), H. Kromhout (the Netherlands) and S. Fuhrimann (South Africa/Netherlands/Switzerland) (both *n* = 9), L. Ezemonye (Nigeria), M. Ishizuka (Japan), K. W. Schramm (Germany), and I. Tongo (Nigeria) (all *n* = 7) (Table S4). In almost two-thirds of the studies, the corresponding author was associated with one out of 106 identified research institutions in Africa (246 (64%)). Most were linked to the University of Cape Town, South Africa (*n* = 19); Makerere University, Uganda (*n* = 12); University of Pretoria, South Africa (*n* = 12); University of Johannesburg, South Africa (*n* = 9); and the University of Benin, Benin (*n* = 8). In addition, 86 research institutions were linked to a corresponding author outside Africa: Hokkaido University, Japan (*n* = 7); Wageningen University, the Netherlands (*n* = 6); Utrecht University, the Netherlands (*n* = 5); Norwegian University of Life Science, Norway (*n* = 4); and University of Antwerp, Belgium (*n* = 4). Leading countries outside Africa were the USA (*n* = 18), the Netherlands (*n* = 17), Norway and Switzerland (both *n* = 13), and France (*n* = 11).

## 4. Discussion

With the observed increased pesticide use in SSA, there is a potential of increasing environmental contamination and acute and chronic health effects after occupational and environmental exposure. This systematic review assessed whether research findings from the region can provide relevant interdisciplinary data for risk reduction and policy interventions. We identified five distinct geographical hot spots where most of the identified research was concentrated: two in South Africa, one in West Africa, and two in East Africa. The major research focus was on insecticides like OCPs and OPs, while health effects of exposure to herbicides and fungicides were seldomly studied, and neither was there a focus on multiple pesticides and their mixtures. Most studies collected environmental samples but only a few environmental risks. Human risk assessments were dominated by model-based risk assessment on measured environmental samples (mostly food and drinking water) and entailed mainly unspecific health risks (e.g., levels above/below maximum residual limits). The few epidemiological studies assessed mostly self-reported symptoms and diseases in small cross-sectional designs. Epidemiological studies applying longitudinal designs, detailed exposure (e.g., biomarkers or algorithms) and health assessments were very limited, even more so limited with a focus on children or women.

The five identified geographic research hotspots in SSA can be described as follows. In the Western Cape in South Africa, the research has been focusing on pesticide use for export-oriented fruit and wheat farming. Most studies were conducted along with the cohort CapSA, which included environmental assessments, exposure, and health assessments of 1000 children living on and off farms [21,22,28,29,30]. In the northeastern region of South Africa [31,32] many studies could be linked to the cohort VHEMBE or the cohort “Occupational health needs of women working on small-scale and emerging farms”, and studies were conducted on pesticide use and its health implications also for woman and their children [33,34]. In East Africa, the PESTROP [24,35,36,37,38,39] and the PEXADU cohorts [25,40] studies in Uganda produced findings on environmental contamination, KAP of pesticide use, and health implications. While other studies focused on water bodies in Kenya [41]. In Ethiopia, several research efforts were conducted around the flower and larger open-farming systems in proximity to the Rift Valley lakes [16,42,43,44,45]. Finally, in West Africa, several connected studies focused on different export-oriented farming systems (e.g., cotton and cocoa) and smallholder farming systems [46]). 

There were 14 articles that spanned across countries. Four studies included even worldwide perspectives of OCPs occurrence in air [47], neonicotinoids environmental and human risks in honey [48], pesticides on wristbands [49], and pesticide-related health effects among smallholder farmers [50]. Two studies assessed KAP of different farming communities [51,52], one on dietary contamination [53], one on fish [54], and three on water [55,56,57]. Seven studies focused on legacy persistent pollutants [58,59] or CUPs in air [30]. Overall, most of these studies focus on past used (legacy) pesticides, while long-term monitoring of CUPs was studied less, and in the case of human exposure (e.g., human biobanks), studies were nonexistent. This makes it difficult to study long-term trends and risks around current agricultural production across SSA.

The focus on OCPs, which are banned for agriculture use, can be explained due to their extensive past use and current use for malaria vector control. Across SSA, most studies found considerable levels of DDT in water, air, soil, human blood [60], or breast milk [61] samples, and its health effects have been reported [62]. The studies raised awareness to reduce human and environmental exposure and possibly a need to reduce the extensions under the Stockholm Convention for vector control. Highly hazardous organophosphates and carbamates were also frequently researched [21,22,63]. Many organophosphates are banned or phased out in high-income countries. Within SSA there are several efforts to regulate these highly hazardous pesticides (HHP) [10], however, with limited success so far [64], as, for example, measured levels in air indicate [30]. There was a critical research gap observed in research on herbicides and fungicides despite herbicide use being the highest of all pesticides in many countries (e.g., South Africa [11]). According to FAO, these two types are currently dominating the total use of pesticides globally and also within SSA [8]. Even though most of the herbicides and fungicides have low acute toxicity, there are often associated with being neurotoxins, endocrine disruptors, or carcinogenic as a result of low-dose long-term exposures [65]. A few of the reviewed studies point out the health risks of herbicides (e.g., glyphosate [35]) and fungicides (e.g., mancozeb [24]). Most studies focused on the one-exposure one-disease approach, taking into account only single effects of pesticides, while several studies investigating use [66,67,68], environmental occurrence [56,69,70,71,72], and human exposure [49,73,74,75] showed that exposure happens from multiple pesticides over time, which could potentially result in cumulative and synergistic environmental [21] and public health effects [24,35]. The missing research on multiple pesticides could be due to the challenges with analyzing these pesticides in local laboratories [76,77] and knowledge gaps with data analysis techniques [35].

Environmental samples were mostly clustered around larger aquatic ecosystems [78,79,80,81,82] and were integrating measurements from water, sediment, or aquatic organisms. Further using passive air [30,47,71,83] and water [21,84,85] sampling systems, several pesticides in the chemical groups of the OCPs and OP, carbamates, and triazine could be detected over several years in different regions across SSA. Indeed, such samplings could define exposure windows over time and space to multiple pesticides and lay a basis for risk assessments. However, among the studies that collected environmental samples, only a few assessed environmental [21,86,87] or human health risks [22,85,88,89,90]. Indeed, in serval cases, there are no contextual environmental risk thresholds available. The studies that applied a risk assessment mostly compared it to EU or U.S. standards. The human health risk assessments were dominated by model-based risk assessment from environmental samples (mostly water [91,92,93] and food [94,95,96]). This might be partly explained by monitoring programs of local water authorities and the focus on export industries, which require testing for residuals in food and flowers. Hence, there are efforts needed to link environmental monitoring campaigns with environmental and human health risks assessments and define context-specific risk thresholds.

The majority of the epidemiological studies were small and assessed self-reported health outcomes cross-sectionally. Only 18 prospective longitudinal studies exist. Out of the 18, in nine objective exposure [25,33,63,97,98,99,100,101,102], in five objective health outcomes [25,63,98,99,103], and in three pesticide dose-response relationship were assessed [25,63,98]. Longitudinal studies provide the best quality evidence but are so scarce that it impacts the quality of evidence for contextual decision making and appropriate mitigation of possible risks. Studies on children and on women were also underrepresented even though they are mostly more vulnerable (e.g., during puberty or pregnancy, respectively) and have different exposure pathways, resulting in different risks linked to the agricultural tasks they perform [43]. To overcome the issue of low-quality evidence, birth cohorts, large biobanks and disease registries should be established across SSA. In addition, health information systems or cohorts in agriculture areas designed to monitor other risks factors (e.g., malaria, HIV) could be considered to co-investigate the risks of pesticides. Moreover, there is a complete absence of data about effective interventions to reduce pesticide use in agriculture (e.g., randomized control trial (RCT) to monitor the effect of training on exposure and risk reduction). The four existing intervention studies were all non-randomized and partly did not compare with a control group [104,105,106,107]. Hence, RCTs applied to intervention studies and targeted to local settings are considered the highest priority for decision making [63].

### Strength and Limitations

Our review has several strengths. That is, we included a search across all pesticide research conducted in 46 countries since over 16 years in two prominent research databases. We also provide a detailed mapping on all 469 study sites, included in the 391 articles. Finally, we provide a comprehensive database in the Supplementary Materials with detailed information on each identified study in terms of the pesticide researched, data collected, and author contributions and contacts (Table S2). Indeed, this database could serve as a networking tool for researchers interested in environmental and human issues around pesticides in SSA to build networks and new collaborations.

A limitation of our review is that some research on pesticides is conducted by governments, NGOs, or the private sector, which do not find their way into the peer-reviewed literature and are therefore not part of this review. Moreover, to limit our search, we excluded studies with a focus on malaria and helminth control. Hence, this could have resulted in missing a few studies that assessed both vector control and agriculture insecticides.

## 5. Conclusions

Conducting a systematic literature review over 16 years, we identified geographical pesticide research hotspots, highlighting that the majority of the studies are on insecticides while researching the most currently used fungicides and herbicides, and their mixtures are largely neglected. The number of identified papers (391) seems to be large. However, most relevant research, such as contextual environmental risk assessments, large cohorts to interpret causality of pesticide-related health risks over time and studies on effective intervention to reduce pesticide exposure, are few or non-existent. Hence, the true picture of pesticide risks for vulnerable SSA ecosystems and populations, such as women, children, adolescents, and immune-compromised who are exposed to pesticides, is not being adequately represented by current studies in the region. Finally, by providing a review of the current state of pesticide research in SSA, we have highlighted the need to increase research and funding for studies to adequately inform risk reduction and pesticide policy interventions to promote sustainable agricultural production in support of the upcoming new green revolution in Africa.

## Figures and Tables

**Table 1:** Most investigated pesticide types and chemical groups across all 469 studies sites (number of study sites (%)).

Investigated Pesticides	All Studies n (%)
Total studies	469 (100)
Assessed pesticide not specified	36 (7.7)
OCPs and CUPs together	101 (21.5)
Only organochlorine pesticides (OCP)	180 (38.4)
Only current-used pesticides (CUP)	152 (32.4)
All OCP	281 (59.9)
OCP (DDT)	241 (85.8)
All CUP	253 (53.9)
All CUP mixtures (I, F, and H together)	68 (26.9)
All insecticides (I)	233 (92.1)
I Organophosphates	180 (77.3)
I Pyrethroids	106 (45.5)
All herbicides (H)	110 (43.5)
All fungicides (F)	89 (35.2)

**Table 2:** Characteristics among the 469 study sites that collected data on human subjects.

Studies including human subjects [study sites n (%)]		180 (38.4)
Overarching topics assessed along with studies with human subjects	Intervention studies (includes also health outcomes)	4 (2.2)
	Health outcomes assessed (can include exposure or KAP)	123 (68.3)
	Only exposure (can include KAP)	49 (27.2)
	Only KAP assessed	4 (2.2)
	Type of study population	
	Occupational	108 (60)
	Environmental	40 (22.2)
	Occupational and environmental	29 (16.1)
	Self-inflicted poisoning	3 (1.7)
	Study design	
	Cross-sectional	151 (83.9)
	Prospective longitudinal	18 (10)
	Retrospective	6 (3.3)
	Case report	5 (2.8)
	Age groups	
	Adults	164 (91.1)
	Adults and children	7 (3.9)
	Children	9 (5)
	Gender of the study population	
	Both gender	111 (61.7)
	Male only	39 (21.7)
	Female only	20 (11.1)
	Gender not assessed	10 (5.6)
	Number of participants (median (range))	
	Cross-sectional studies	183 (7–1496)
	Prospective longitudinal studies	310 (15–1461)
	Retrospective studies	797 (96–7427)
All health outcomes assessments	All health outcomes	127 (70.6)
	Analysis method of outcomes	
	Regression model to assess exposure/outcome	36 (28.3)
	Significant positive associations observed	28 (77.8)
	Descriptive reporting of outcomes	77 (60.6)
	Model-based risks	14 (11)
	Self-reported signs and symptoms of acute poisoning	38 (29.9)
	Doctor-diagnosed pesticide poisoning	24 (18.9)
	Neurological assessment	17 (13.4)
	Reproductive health	14 (11)
	Respiratory health	11 (8.7)
	Unspecific human health risks	12 (9.4)
	Kidney and/or liver problems	6 (4.7)
	Other: Diabetes, hypertension, cancer	1 (0.8)
	Assessment method	
	Self-reported	58 (45.7)
	Objective measures	41 (32.3)
	Doctor-diagnosed	16 (12.6)
	Model-based risks	12 (9.4)
Human exposure assessments	All human exposure assessments	171 (95)
	Objective exposure markers and self-reported exposure data	45 (26.3)
	Only objective exposure markers	15 (8.8)
	Exposure algorithms based on self-reported data	4 (2.3)
	Only self-reported exposure data	107 (62.6)
	All matrices objective human exposure markers were assessed	60 (35.1)
	Blood	36 (60)
	Urine	14 (23.3)
	Wristbands	5 (8.3)
	Breast milk	4 (6.7)
	Other	5 (8.3)
KAP	All knowledge, attitude, and practice (KAP)	149 (82.8)
	Training on pesticide use	40 (26.8)

**Table 3:** Characteristics among the 469 study sites that collected environmental samples.

All environmental samples [study sites n (%)]		317 (67.6)
Integrative assessment	1 matrix	217 (68.4)
	2 matrices	55 (17.4)
	3 or more matrices	45 (14.2)
Individual matrices collected (overlaps between matrices)	Water	93 (29.4)
	Aquatic species	79 (25)
	Sediment	73 (23.1)
	Agricultural produce	72 (22.8)
	Air	57 (18)
	Soil	39 (12.3)
	Other matrices	34 (10.4)
	Dust	2 (0.6)
Envrionmental risk assessment (among collected samples)	All environmental risk assessments	89 (28.2)
	Levels of at least one pesticide above risk threshold	76 (85.4)
	Water	41 (44.1)
	Sediment	33 (45.2)
	Aquatic species	27 (34.2)
	Agricultural produce	24 (33.3)
	Other matrices	14 (42.4)
	Soil	9 (23.1)
Human health risk assessment	All human health risk assessments	112 (35.4)
	Levels of at least one pesticide above risk threshold	53 (47.3)
	Human health outcomes investingated	
	Unspecific human health risks	78 (69.6)
	Cancer	14 (12.5)
	Pesticide poisoning	13 (11.6)
	Reproductive health	5 (4.5)
	Neurological assessment	2 (1.8)
	Exposure pathways	
	Consumption	107 (95.5)
	Ingestion	2 (1.8)
	Inhalation	3 (2.7)

## Data Availability

Data extracted from the 391 identified articles are provided in the Supplementary Materials.

## Supplementary Materials

The following are available online at https://www.mdpi.com/article/10.3390/ijerph19010259/s1, Table S1. Search terms and hits in Pubmed and Web of Science (WoS); Table S2. Database-extracted 469 study sites; Table S3. Summary (n (%)) of the 469 studies’ collected primary data around pesticides stratified by the 37 identified countries where the studies were conducted; Table S4. Showing list of authors who contributed as leading authors (first, second or last) to at least three of the 391 publications.
