# Supplementary material for: Pesticide Research on Environmental and Human Exposure and Risks in Sub-Saharan Africa: A Systematic Literature Review
Source: Int J Environ Res Public Health. 2021 Dec 27;19(1):259. doi: 10.3390/ijerph19010259 (PMC8750985; doi:10.3390/ijerph19010259)
Supplement: Supplementary file 1 [file ijerph-19-00259-s001.zip › ijerph-1469778-supplementary/SI information.pdf]

Pesticide Research on Environmental and Human Exposure and Risks in Sub-Saharan Africa: A Systematic Literature Review

Samuel Fuhrimann [1,2,4,*], Chenjie Wan [3], Elodie Blouzard [4], Adriana Veludo [1,2], Zelda Holtman [6,7], Shala Chetty-Mhlanga [1,2], Mohamed Aqiel Dalvie [6], Aggrey Atuhaire [5], Hans Kromhout [4], Martin Röösli [1,2], and Hanna-Andrea Rother [6,7]

[1] Swiss Tropical and Public Health Institute (Swiss TPH), 4002, Basel, Switzerland; samuel.fuhrimann@swisstph.ch; adrianaveludo@gmail.com (A.V.); shalachetty7@gmail.com (S.C.-M.); martin.roosli@swisstph.ch (M.R.)
[2] University of Basel, 4002, Basel, Switzerland
[3] Department of Environmental Systems Science, ETH Zurich, Zurich, Switzerland; chenjie_wan@outlook.com (W.C.)
[4] Institute for Risk Assessment Sciences (IRAS), Utrecht University, 3584 CM, Utrecht, The Netherlands; elodie.blouzard96@gmail.com (E.B.); h.kromhout@uu.nl (H.K.)
[5] Uganda National Association of Community and Occupational Health (UNACOH), 12590, Kampala, Uganda;atuagrey3@gmail.com (A.A.)
[6] Centre for Environmental and Occupational Health Research, School of Public Health and Family Medicine, University of Cape Town, 7729, Cape Town, South Africa; aqiel.dalvie@uct.ac.za (M.A.D.); andrea.rother@uct.ac.za (H.-A.R.); zeldaholtman57@gmail.com(Z.H.)
[7] Division of Environmental Health, School of Public Health and Family Medicine, University of Cape Town, 7729, Cape Town, South Africa

* Correspondence: samuel.fuhrimann@unibas.ch

Table S1. Search terms and hits in Pubmed and Web of Science (WoS).

Broader search citeria	Key words	Search terms hits	
		**Pubmed**	**WoS**
**Search time window**	/01/01"[PDat] : "2021/26/10)"[PDat])		
**Pesticides**<br>**(11 terms)**	pesticid* OR “plant protection product*” OR PPP OR herbicid* OR fungicid* OR insecticid* OR acaricid* OR nematicid* OR molluscicid* OR rodenticide* OR biocid*	141,262	215,941
**AND**<br>**46 countires in Sub-Shaharan Africa**<br>**(66 terms)**	africa*<br>OR angola* OR benin* OR botswana OR "burkina faso" OR "upper volta" OR burundi<br>OR "côte d’ivoire" OR "cote d’ivoire" OR "ivory coast"<br>OR cameroon OR cameroun OR kameru<br>OR "central african republic" OR chad OR congo OR zaire OR djibouti OR "equatorial guinea" OR eritrea OR ethiopia OR gabon OR gambia OR guinea OR guinea-bissau OR kenya OR lesotho OR liberia OR malawi OR mali OR mauritania OR mozambique OR namibia OR niger* OR rwanda OR senegal OR "sierra leone" OR somalia OR "south africa" OR "south sudan" OR sudan OR swaziland OR tanzania OR togo OR uganda OR zambia OR zimbabwe OR Rhodesia OR "cabo verde" OR comoros OR congo* OR gambia OR madagascar OR mauritius OR "sao tome and principe" OR seychelles OR abyssinia OR biafra OR ruanda OR sahel OR urundi OR zaire OR zanzibar OR ghana OR maghreb OR libya	8,868	13924
**AND Human subjects** – **Objective markers exposure**<br>**(21 terms)**	biomarker* OR metabolite* OR urine OR urinary OR *nail* OR blood OR acetylcholinesterase OR AChE OR hair OR tissue* OR skin OR dermal OR patch* OR sweat OR spit OR swab*OR stool OR feces OR faeces OR milk OR wristband*	2,438	2,579
**Qualitative exposure**<br>**(5 terms)**	exposure* OR KAP OR knowledge OR attitude* OR practice* OR questionnaire*	2,902	4,151
**Exposoure groups**<br>**(9 terms)**	job OR operator* OR applicator* OR farm* OR applicator* OR work* OR mother* OR *children* OR veterinarian*	2,612	6,343
**AND Enviromental samples (7 terms)**	residual* OR food OR water OR air OR soil* OR sediment* OR dust*	3,366	6,113
**All togehter**	(pesticid* OR “plant protection product*” OR PPP OR herbicid* OR fungicid* OR insecticid* OR acaricid* OR nematicid* OR molluscicid* OR rodenticide* OR biocid*)<br>AND<br>(*africa* OR angola* OR benin* OR botswana OR "burkina faso" OR "upper volta" OR burundi OR "côte d’ivoire" OR "cote d’ivoire" OR "ivory coast" OR cameroon OR cameroun OR kameru OR "central african republic" OR chad OR congo OR zaire OR djibouti OR "equatorial guinea" OR eritrea OR ethiopia OR gabon OR gambia OR guinea OR guinea-bissau OR kenya OR lesotho OR liberia OR malawi OR mali OR mauritania OR Mozambique OR namibia OR niger* OR rwanda OR senegal OR "sierra leone" OR somalia OR "south africa" OR "south sudan" OR sudan OR swaziland OR tanzania OR togo OR uganda OR zambia OR zimbabwe OR Rhodesia OR "cabo verde" OR comoros OR *congo* OR *gambia OR madagascar OR mauritius OR "sao tome and principe" OR seychelles OR abyssinia OR biafra OR ruanda OR sahel OR urundi OR zaire OR zanzibar OR ghana OR maghreb OR libya)<br>AND<br>((pesticid* OR “plant protection product*” OR PPP OR herbicid* OR fungicid* OR insecticid* OR acaricid* OR nematicid* OR molluscicid* OR rodenticide* OR biocid*)<br>OR (biomarker* OR metabolite* OR urine OR urinary OR nail* OR blood OR hair OR tissue* OR skin OR dermal OR patch* OR sweat OR spit OR swab*OR stool OR feces OR faeces OR milk OR wristband*<br>OR job OR operator* OR applicator* OR farm* OR applicator* OR work* OR mother* OR children* OR veterinarian*<br>OR exposure* OR KAP OR knowledge OR attitude* OR practice* OR questionnaire*<br>OR residual* OR food OR water OR air OR soil* OR sediment* OR dust*))	6,731	11,106
**Exclusion**	NOT malaria NOT helminth*	4,027	6,443

Table S2. database - extracted 469 study sites.

See separate excel data base include all extracted data from 464 study sites.

Table S3 Summary (n (%)) of the 469 study sites collected primary data around pesticides stratified by the 37 indentified country where the studies were conducted.

Country	Total Study sites	Eniron-mental samples	Human exposure	Human health	KAP	Inter-vention studies	OCP	CUP
Total	469 (100)	316 (67.4)	171 (36.5)	229 (48.8)	149 (31.8)	4 (0.9)	281 (59.9)	253 (53.9)
South Africa	102 (21.7)	72 (15.4)	36 (7.7)	44 (9.4)	30 (6.4)	1 (0.2)	63 (13.4)	51 (10.9)
Nigeria	45 (9.6)	37 (7.9)	10 (2.1)	25 (5.3)	10 (2.1)	0 (0)	32 (6.8)	18 (3.8)
Ethiopia	42 (9)	23 (4.9)	22 (4.7)	27 (5.8)	22 (4.7)	0 (0)	28 (6)	20 (4.3)
Uganda	37 (7.9)	18 (3.8)	20 (4.3)	21 (4.5)	16 (3.4)	2 (0.4)	16 (3.4)	23 (4.9)
Tanzania	37 (7.9)	24 (5.1)	15 (3.2)	24 (5.1)	13 (2.8)	0 (0)	30 (6.4)	19 (4.1)
Ghana	32 (6.8)	13 (2.8)	19 (4.1)	20 (4.3)	16 (3.4)	0 (0)	13 (2.8)	22 (4.7)
Kenya	26 (5.5)	23 (4.9)	3 (0.6)	9 (1.9)	3 (0.6)	0 (0)	10 (2.1)	16 (3.4)
Benin	19 (4.1)	13 (2.8)	6 (1.3)	10 (2.1)	6 (1.3)	0 (0)	15 (3.2)	10 (2.1)
Cameroon	17 (3.6)	6 (1.3)	10 (2.1)	9 (1.9)	10 (2.1)	0 (0)	5 (1.1)	12 (2.6)
Senegal	12 (2.6)	8 (1.7)	5 (1.1)	4 (0.9)	3 (0.6)	0 (0)	9 (1.9)	8 (1.7)
Togo	9 (1.9)	7 (1.5)	2 (0.4)	3 (0.6)	2 (0.4)	0 (0)	7 (1.5)	3 (0.6)
Sudan	8 (1.7)	6 (1.3)	2 (0.4)	1 (0.2)	1 (0.2)	0 (0)	7 (1.5)	5 (1.1)
Mali	8 (1.7)	7 (1.5)	2 (0.4)	1 (0.2)	2 (0.4)	1 (0.2)	5 (1.1)	3 (0.6)
Burkina Faso	8 (1.7)	5 (1.1)	4 (0.9)	5 (1.1)	3 (0.6)	0 (0)	6 (1.3)	8 (1.7)
Zambia	7 (1.5)	6 (1.3)	1 (0.2)	0 (0)	1 (0.2)	0 (0)	6 (1.3)	3 (0.6)
Zimbabwe	6 (1.3)	2 (0.4)	4 (0.9)	3 (0.6)	2 (0.4)	0 (0)	2 (0.4)	4 (0.9)
Niger	6 (1.3)	5 (1.1)	1 (0.2)	3 (0.6)	1 (0.2)	0 (0)	3 (0.6)	4 (0.9)
Mozambique	6 (1.3)	5 (1.1)	1 (0.2)	2 (0.4)	1 (0.2)	0 (0)	3 (0.6)	3 (0.6)
DR Congo	5 (1.1)	5 (1.1)	0 (0)	1 (0.2)	0 (0)	0 (0)	4 (0.9)	1 (0.2)
Côte D'ivoire	5 (1.1)	3 (0.6)	2 (0.4)	5 (1.1)	1 (0.2)	0 (0)	1 (0.2)	3 (0.6)
Congo	5 (1.1)	5 (1.1)	1 (0.2)	1 (0.2)	1 (0.2)	0 (0)	4 (0.9)	1 (0.2)
Botswana	4 (0.9)	3 (0.6)	1 (0.2)	0 (0)	1 (0.2)	0 (0)	2 (0.4)	2 (0.4)
Mauritius	3 (0.6)	3 (0.6)	0 (0)	0 (0)	0 (0)	0 (0)	2 (0.4)	1 (0.2)
Malawi	3 (0.6)	3 (0.6)	0 (0)	3 (0.6)	0 (0)	0 (0)	0 (0)	3 (0.6)
Swaziland	2 (0.4)	1 (0.2)	1 (0.2)	1 (0.2)	0 (0)	0 (0)	1 (0.2)	1 (0.2)
Rwanda	2 (0.4)	2 (0.4)	0 (0)	0 (0)	1 (0.2)	0 (0)	1 (0.2)	1 (0.2)
Madagascar	2 (0.4)	2 (0.4)	0 (0)	2 (0.4)	0 (0)	0 (0)	0 (0)	2 (0.4)
Guinea	2 (0.4)	2 (0.4)	0 (0)	1 (0.2)	0 (0)	0 (0)	1 (0.2)	2 (0.4)
Somalia	1 (0.2)	1 (0.2)	0 (0)	0 (0)	0 (0)	0 (0)	1 (0.2)	0 (0)
Sierra Leone	1 (0.2)	0 (0)	1 (0.2)	1 (0.2)	1 (0.2)	0 (0)	0 (0)	1 (0.2)
Seychelles	1 (0.2)	1 (0.2)	0 (0)	0 (0)	0 (0)	0 (0)	1 (0.2)	0 (0)
Mauritania	1 (0.2)	1 (0.2)	0 (0)	0 (0)	0 (0)	0 (0)	1 (0.2)	1 (0.2)
Lesotho	1 (0.2)	0 (0)	1 (0.2)	1 (0.2)	1 (0.2)	0 (0)	0 (0)	0 (0)
Guinea-Bissau	1 (0.2)	1 (0.2)	1 (0.2)	0 (0)	1 (0.2)	0 (0)	1 (0.2)	0 (0)
Eritrea	1 (0.2)	1 (0.2)	0 (0)	1 (0.2)	0 (0)	0 (0)	0 (0)	1 (0.2)
Chagos	1 (0.2)	1 (0.2)	0 (0)	0 (0)	0 (0)	0 (0)	1 (0.2)	0 (0)
Central African Republic	1 (0.2)	1 (0.2)	0 (0)	1 (0.2)	0 (0)	0 (0)	0 (0)	1 (0.2)

CUP: current-used pesticide; OCP: organocholorine pesticides; KAP: knowledge, attitude and practice

Table S4 Showing list of authors which contributed as leading authors (firs, second or last) to at least three of the 391 publications.

Author lastname	Number of occurance
Dalvie	14
London	14
Bouwman	10
Fuhrimann	9
Kromhout	9
Ezemonye	7
Ishizuka	7
Schramm	7
Tongo	7
Bornman	6
Ikenaka	6
Jors	6
Sosan	6
Wepener	6
Abdelbagi	5
Barnhoorn	5
Lalah	5
Naidoo	5
Ngowi	5
Okoh	5
Otieno	5
Polder	5
Spanoghe	5
Yohannes	5
Akoto	4
Atabila	4
Chu	4
Eskenazi	4
Ikpesu	4
Kiremire	4
Lyche	4
Mahugija	4
Mdegela	4
Mekonen	4
Müller	4
Schulz	4
Ssebugere	4
Torto	4
Yehouenou	4

Author lastname	Number of occurance
Akingbohungbe	3
Azehoun	3
Ball	3
Bervoets	3
Bollmohr	3
Chimuka	3
de Jager	3
Deribe	3
Eklo	3
Gerber	3
Humphries	3
Kishimba	3
Laing	3
Lehmann	3
Lekei	3
Moen	3
Mutengwe	3
Mwevura	3
Negatu	3
Ogbeide	3
Ojemaye	3
Onwordi	3
Owuor	3
Oyekunle	3
Petrik	3
Phung	3
Poté	3
Rosseland	3
Rother	3
Schlünssen	3
Sichilongo	3
Skaare	3
Smit	3
Teklu	3
Van den Brink	3
Vermeulen	3
Wasswa	3
